# Squid Skin Decellularised Dermal Matrix for Enhancing Repair of Acute Cranial Injuries in Rabbit Model

**DOI:** 10.3390/jfb16050159

**Published:** 2025-04-30

**Authors:** Lixin Liu, Yida Pang, Haoze Yang, Qiyi Zhou, JinHua Hou, Wenhui Wu, Jeevithan Elango

**Affiliations:** 1 Department of Marine Biopharmacology, College of Food Science and Technology, Shanghai University, Shanghai 201306, China; 18651125617@163.com (L.L.); 2334219@st.shou.edu.cn (Y.P.); yhz990328@163.com (H.Y.); zhouqiyi_971@yeah.net (Q.Z.); levousaime@163.com (J.H.); 2 Putuo Sub-Center of International Joint Research Center for Marine Biological Sciences, Zhoushan 316104, China; 3 Center of Molecular Medicine and Diagnostics (COMManD), Department of Biochemistry, Saveetha Dental College and Hospitals, Saveetha Institute of Medical and Technical Sciences, Saveetha University, Chennai 600077, India; 4 Department of Biomaterials Engineering, Faculty of Health Sciences, UCAM Universidad Católica San Antonio de Murcia, 30107 Murcia, Spain

**Keywords:** squid skin, decellularised dermal matrix, collagen, cranial injury, biomedical material

## Abstract

Squid skin decellularized dermal matrix (SADM) is gaining attention in tissue engineering and regenerative medicine due to its mimicking of the extracellular matrix property. Hence, SADM was used to investigate mimicking the microenvironment of cellular growth, inducing cellular infiltration and angiogenesis, and facilitating the repair of acute craniofacial wounds. For this, tissue regeneration membranes from squid skin were prepared by decolorization, degreasing and decellularisation methods. The effect of SADM in guiding bone tissue regeneration was evaluated using the rabbit skull bone defect model. SEM images of SADM had a bilayer membrane architecture characterized by a reticulated porous structure on one side and a dense, non-porous surface on the opposite side. Notably, the water absorption capacity of SADM was approximately eight times higher than its weight, exhibiting a porosity of 58% and a peak average tensile stress of 10.43 MPa. Additionally, simulations of tissue fluid degradation indicated a degradation rate of 70.42% and 88.33% on days 8 and 12, respectively. Following 4 and 8 weeks of animal studies focused on repairing cranial bone defects in rabbits, the findings demonstrated that SADM served as an effective barrier against fibrous connective tissue, promoted the proliferation of osteoblasts, and supported bone regeneration. This was confirmed through micro-CT imaging, and sections were stained with senna solid green. In summary, SADM is capable of directing cell infiltration and bone tissue formation, modulating the expression and secretion of inflammatory and skin repair-related factors, thereby enhancing tissue healing.

## 1. Introduction

Bone plays a crucial role in various life activities, serving not only as a structural framework but also as a vital component in movement and overall health. Unfortunately, many patients suffer from orthopedic diseases resulting from traffic accidents and workplace injuries, leading to significant challenges in treatment [1,2]. The destruction of bone structure and integrity often results in extensive tissue defects that are difficult to heal independently. This can severely impair limb function due to bone loss and nonunion, potentially leading to lifelong disabilities. While advancements in techniques such as osteoconduction, distraction osteogenesis, and osteoinduction have made strides in addressing bone repair, clinical applications still face numerous limitations. These include heightened risks of secondary damage and complications at the healing site, extended treatment durations, and increased costs [3,4,5,6]. A landmark development occurred in 1989 when Dahlin introduced the guided bone tissue regeneration technique for immediate implantation and guided peri-implant bone regeneration, achieving promising results in bone repair [7]. The recent proliferation of this technique has opened new avenues for treating bone defects, offering innovative solutions to longstanding challenges in orthopedic care.

Collagen is a fundamental component of the extracellular matrix (ECM), playing a crucial role in tissue structure and function [8,9,10,11]. Among the twenty-nine identified types of collagen, type I collagen is prevalent in various tissues, including skin, tendons, and bones [12,13]. Its remarkable biological properties, like biocompatibility, biodegradability, and low immunogenicity, facilitate the directed growth of osteoblasts and other cells [14,15,16], making it a vital material in biomedical tissue engineering. As the demand for biomedical materials increases, heterologous collagen sourced from terrestrial mammals like pigs and cattle has gained popularity [17]. However, concerns regarding zoonotic diseases, such as foot-and-mouth disease and mad cow disease, alongside high production costs, pose significant challenges in meeting this demand [18,19]. Consequently, extracting collagen from marine organisms emerges as a promising alternative for developing collagen biomaterials [20,21,22,23].

Fish skin decellularised dermal matrix (ADM) is produced by extracting collagen in an alkaline solution, followed by decellularisation, which removes the cellular structures of animal skin tissues [24,25,26]. This process leaves behind a relatively less immunogenic ECM and a collagen fiber mesh that serves as a cellular scaffolding structure. This matrix is capable of providing and maintaining tissue structure while guiding and regulating cell growth and migration, thus playing a pivotal role in skin repair processes [27,28]. Currently, it is widely utilized in various biomedical applications, including acute skin trauma treatment, bone regeneration, and dura repair, receiving positive feedback in clinical settings [29,30].

Research on fish collagen has expanded significantly. For instance, studies have demonstrated that decellularised fish skin outperforms decellularised cow skin in burn repair [31]. Additionally, collagen extracted from tilapia has shown rapid wound-healing capabilities in rat models, while marine collagen peptides derived from tilapia skin have been effective in promoting healing for second-degree burns [32]. Furthermore, electrospun nanofibers from tilapia skin collagen have been shown to induce skin wound healing [33]. These findings suggest that collagen-rich fish skin materials possess high biodegradability, indicating a promising application for squid skin decellularised dermal matrix (SADM) in acute wound repair and regenerative medicine [34,35]. Squid, being a significant contributor to marine fish catches, offers advantages such as high availability, rapid growth rates, and cost-effectiveness compared to tilapia [36].

In this study, we prepared SADM from squid skin and evaluated its physicochemical properties. We employed co-culture and implantation methods to assess its biodegradability and biocompatibility, comparing it with commercially available fetal bovine-derived periosteum (FBADM). Additionally, we conducted acute cranial trauma repair experiments on rabbits to further verify the biological properties of SADM.

## 2. Materials and Methods

### 2.1. Materials

Squid was purchased from Haixin Aquatic Products Co., Fuzhou, China. HEAL-ALL membranes are prepared from bovine hides after a series of treatments with an allogeneic decellularised dermal matrix, whose main ingredient is collagen, and are radiation sterilized for single use. (Yantai Zhenghai Bio-Tech, Yantai, China). All other chemicals were of analytical grade and could be used without further purification. Six New Zealand white rabbits (12–14 weeks, mean weight: 2.5–3.5 kg) were used in this animal experiment. This study was approved by the Institutional Animal Care and Use Committee of Shanghai Ocean University (ACUC Approval Number: SHOU-DW-2024-140).

### 2.2. Squid Decellularised Dermal Matrix Preparation (SADM)

The treated squid skin was soaked in a 3% sodium bicarbonate (NaHCO_3_) solution for 12 h at a material-liquid ratio of 1:10 (*w*/*v*). Fish skin was soaked in 0.1 M NaOH solution for 8 h at a material-liquid ratio of 1:10 (*w*/*v*). The fat and other impurities of squid skins were removed by 0.01 M phosphate buffer solution (PBS) wash. The fish skin was then soaked in 1 M NaCl and 0.1 M NaOH at a material-liquid ratio of 1:5 and 1:10 (*w*/*v*) for 12 and 6 h, respectively. The squid skin was bleached by soaking in 3% hydrogen peroxide (H_2_O_2_) solution with a feed liquid ratio of 1:5 (*w*/*v*) for 6 h, followed by 0.01 M PBS wash to decellularise the fish skin. Finally, SADM (wet) was washed with purified water, cut into 5 cm × 5 cm sizes, and freeze-dried.

### 2.3. Physicochemical Properties of SADM

#### 2.3.1. HE Staining

SADM was embedded in paraffin, dried in sections at 60 °C, and incubated in xylene; sections were immersed in hematoxylin stain for 5 min, rinsing excess stain under running water, 1% hydrochloric acid alcohol immersion for 10 s and rinsing under running water for 15 min [37,38]. The sections were differentiated with 1% hydrochloric acid ethanol by volume, re-stained with 0.5% eosin for 3 min, transparent with xylene, accelerated curing in a 37 °C oven and then sealed with neutral gum, and observed under a microscope.

#### 2.3.2. Massion Staining

SADM was fixed with 4% (*w*/*v*) paraformaldehyde, dehydrated and embedded in paraffin, as previously mentioned. Sections were made to a thickness of 5 μm, stained with Weigert’s iron hematoxylin for 5 min and rinsed. The sections were treated with 1% phosphomolybdic acid solution for 5 min and stained with Masson staining solution for 5 min. The slices were accelerated to cure in an oven at 37 °C and then sealed with neutral gum and observed microscopically [39].

#### 2.3.3. Microstructure Observation

The structure of SADM was observed by SEM. In brief, the SADM was cut into small pieces of 1 mm × 1 mm and fixed on the sample stage with conductive adhesive tape. The microstructure of the SADM surface after gold spraying was observed using a Smur4800 cold field emission scanning electron microscope (ZEISS Sigma 300, Thuringia, Germany).

#### 2.3.4. Porosity

The porosity of the membranes was investigated by measuring the changes in the material volume after treatment with ethanol for solution substitution [40]. Dry samples were fully submerged in a plastic container filled with anhydrous ethanol, initially measured at a volume of *W*1. The ethanol was degassed until no bubbles were visible, and the volume at this stage was recorded as *W*2. After removing the samples, the remaining ethanol volume was noted as *W*3. Three samples from each group were measured, and the average value was calculated. Porosity was determined using the following formula:

$$\theta \;(\%)=\frac{W1-W3}{W2-W3}\times 100\%$$

#### 2.3.5. Dissolution Properties

The swelling rate is an important indicator for evaluating the hydrophilicity of a dressing, as higher swelling rates favor cell adhesion and osmotic growth [41,42,43,44]. Rectangular samples, fully dried and measuring 2 × 2 cm, were precisely weighed as *Wd*. These samples were then immersed in ultrapure water, saline, and SPF-simulated tissue solution for durations of 0.5 h, 2 h, 12 h, 24 h, and 48 h. After immersion, excess surface water was removed using filter paper until no liquid dripped, and the samples were weighed again as *Ws*. Four samples were analyzed in each group, and the average value was recorded. The water absorption of the samples was determined using the specified formula:

$$W\;(\%)=\frac{Ws-Wd}{Wd}\times 100\%$$

#### 2.3.6. Fourier Infrared Spectroscopy

The SADM was sectioned into 1 mm × 1 mm pieces, then crushed and utilized for infrared spectroscopy analysis. The infrared spectra of collagen within the scaffolds were obtained using Fourier transform infrared spectroscopy (Nicolet 6700, Thermo Fisher, Hopkinton, MA, USA), covering a scanning range of 400–4000 cm^−1^. Data analysis was performed using Origin software (version 2021 MicroCal, Northampton, MA, USA). The identified absorption peaks for collagen included amide A, amide B, amide I, amide II, and amide III. The secondary structures of the collagens were examined in the 1600–1700 cm^−1^ range of the amide I region, employing PeakFit version 4.12 software (SeaSolve Software Inc., Framingham, MA, USA) and the Gaussian peak fitting method. The percentage of each secondary structure was determined by calculating the ratio of the peak area of each structure to the total peak area of all secondary structures.

#### 2.3.7. Determination of Mechanical Properties

To evaluate the mechanical strength of the brackets, SADM with a thickness of 0.6 mm (measured with a spiral micrometer) was cut into 3.5 cm × 0.2 cm strips at room temperature and tested for tensile strength using a general-purpose materials testing machine (CMT8502, MTS Systems, Jinhua, China). The samples were placed in a fixture with a maximum load of 500 cN, a sensitivity of 0.01 cN, and a test speed of 5 mm/min. The number of repetitions for all the tests was 5, and the average value was taken.

### 2.4. Determination of In Vitro Degradation Rate

A crucial approach to evaluating the degradation performance and structural integrity of membranes involves assessing their in vitro degradation rate [45,46]. Under conditions simulating lysozyme degradation, the membranes exhibited degradability, characterized by mild swelling, thinning, and dissolution within the degradation solution. A lysozyme solution at a concentration of 30 U/mL was prepared using PBS buffer (pH 7.4) as the degradation medium. A sample measuring 1.5 × 2.0 cm of UV-sterilized material was placed in a test tube, followed by the addition of 20 mL of the prepared degradation solution to ensure complete submersion of the sample. The test tubes were then sealed and incubated at 37 °C. Samples were collected and stored on days 2, 4, 6, 8, 10, and 12. The remaining undegraded scaffold material was lyophilized and weighed, with the experiment being repeated three times to calculate the degradation rate using the appropriate formula:

$$D\;(\%)=\frac{W1-W2}{W1}\times 100\%$$

where D is the degradation rate, W1 is the weight before degradation, and W2 is the weight after degradation.

### 2.5. Cell Culture

To assess the cytotoxicity and proliferation rate, human bone marrow-derived MSCs (Meisen-CTCC, Jinhua, China) were cultured on SADM. Previously, MSCs were cultured on T75 culture flasks with DMEM medium supplemented with 10% FBS and 1% antibiotics. Upon confluence, the cells were trypsinized and used for further process. The cells from passages 3–8 were used for the following experiments.

#### Cytotoxicity and Proliferation Rate

Deionized water served as the extraction medium, and the sterilized scaffold material was sectioned into 10 × 10 mm blocks. The scaffold material was combined with DMEM liquid medium to create an extraction solution at a concentration of 1 mg/mL. Both material groups were macerated at 37 °C for 24 ± 1 h. Following this, the macerated solution was centrifuged, and the supernatant was collected for further analysis [47,48].

Cytotoxicity was assessed using DAPI staining and the CCK-8 assay. Once the cells reached the logarithmic growth phase, they were counted using a cell counter (TC20, BioRad, Hercules, CA, USA). A volume of 200 μL of cell suspension was dispensed into 96-well plates, adhering to the standard of 1 × 10^5^ cells per well. The control group consisted of three parallel samples, while the experimental group also included three parallel samples. After the cells adhered to the plate, the original culture medium was replaced with the extraction solution. Following a 15-min incubation at 37 °C, the cells were double-stained with DAPI according to the kit instructions (Solarbio, Shanghai, China) and incubated at 37 °C for SADM. An inverted fluorescence microscope (ECHO RVL-100-G, San Diego, CA, USA) was employed to excite fluorescence at a wavelength of 490 nm, allowing for the observation of live and dead cell distribution. According to the standard of 5 × 10^3^ cells per well, 200 μL of the mixture of cell suspension and extract was added to the 96-well plate in sequential ratios of 20%, 40%, 60%, 80%, and 100%. After co-culturing with the cells for 24 h, 100 μL of CCK-8 solution was introduced to each well, following the kit instructions (10 μL of CCK-8 in 90 μL of medium). The plates were then incubated at 37 °C with 5% CO_2_ for 2 h, and the optical density (OD) at 470 nm was measured using a microplate reader (Epoch, BioTek, Winooski, VT, USA).

### 2.6. Rabbit Skull Injury Model

#### 2.6.1. Establishment and Surgical Repair of Cranial Parietal Hole-Type Bone Defects in the Rabbit

Six New Zealand white rabbits (12–14 weeks, mean weight: 2.5–3.5 kg) were used in this animal experiment. This study was approved by the Institutional Animal Care and Use Committee of Shanghai Ocean University (ACUC Approval Number: SHOU-DW-2024-140). Under general anesthesia, the rabbit cranial parietal coat (range ≥ 3 × 3 cm) was shaved and disinfected, and a sterile cavity towel was spread to establish the operating field. The skin and subcutaneous tissue were incised longitudinally along the junction of the sagittal suture and the coronal suture for about 2.5 cm using a surgical blade, and the periosteum was peeled off in a circular fashion to fully expose the midline region of the parietal bone. A high-speed diamond drill (6.0 mm diameter, 10,000 rpm) was used to prepare three 6-mm round full-layer bone defects with the dura intact, and the drilling process was stopped when the internal and external bony plates were penetrated to the surface of the dura, and the bone debris was rinsed out thoroughly with continuous saline rinsing to avoid thermal damage. Differential biofilm materials were implanted precisely according to subgroup requirements [49,50]. The areas of the bone defects were covered with SADM (experimental group) and HEAL-ALL periosteum (positive control group) and were treated with no coverage of the membranes (negative control group), and periosteal sutures were placed on the experimental group and the positive control group, respectively. Gentamicin was injected postoperatively once a day for 3 d to prevent infection. New Zealand White rabbits were euthanized with an overdose of sodium pentobarbital (150 mg/kg, intramuscular injection) at 4 and 8 weeks postoperatively, and cranial specimens were obtained from the area of the defect for the relevant tests. And then immersed in 10% formalin for 7 days.

#### 2.6.2. Observation Indicators

General observations included the assessment of post-operative diet, activity levels, and wound healing in New Zealand Large White rabbits following the experiment. For imaging, micro-CT scans were conducted on the cranial parietal bone specimens of the experimental animals, which were euthanized at 4 and 8 weeks post-operation. The histological analysis involved collecting and staining samples from the repair site with Senna green after the rabbits were euthanized, allowing for the examination of new bone tissue formation under a light microscope.

#### 2.6.3. Statistical Analysis

The statistical analysis was undertaken using statistical software (SPSS ver. 25.0, IBM Corp, Chicago, IL, USA). The in vitro results were analyzed by one-way ANOVA followed by Bonferroni’s post hoc U test. In the in vivo study, the Kruskal–Wallis test followed by the Mann–Whitney U post hoc test was performed to compare the results of new bone volume; the one-way ANOVA followed by the Bonferroni post hoc test was carried out for the histometric result. The significance of differences was accepted for *p*-value < 0.05.

## 3. Results and Discussion

### 3.1. Histology

**Figure 1:**
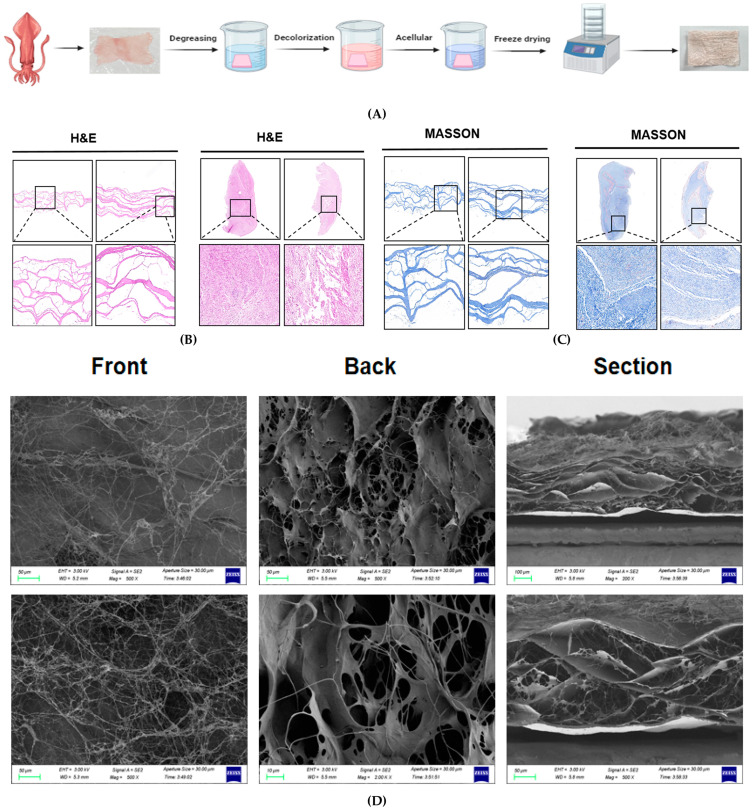
Preparation and characterization of SADM. (**A**) flow chart of SADM preparation; (**B**) H&E staining map of squid skin before and after acellular treatment; (**C**) MASSON staining map of squid skin before and after acellular treatment; (**D**) SADM scanning electron microscope.

The results of HE staining on the decellularized membrane matrix revealed that the matrix membranes, following the decellularization process, consisted entirely of collagen fibers arranged in pink bands, with no blue indicators present. This finding suggests that the cellular components of the fish skin were effectively eliminated, leaving only the collagen matrix intact. The fibers were systematically organized into bundles, resulting in a more loosely structured tissue (Figure 1B).

The results of Masson staining indicated that the decellularized membrane matrix from the squid lacked red cytoplasm, dark blue-black nuclei, and other substances that were stained blue. Additionally, the collagen fibers were organized in a specific direction, exhibiting no disorder or interruption (Figure 1C). This observation suggests that the structural integrity of collagen fibers was preserved following the decellularization process.

### 3.2. Scanning Electron Microscopy

The micro-morphological SEM results showed that following the decellularization process, the subcutaneous tissue was effectively eliminated, resulting in an epidermal surface that exhibited a more porous and relatively uniform lax texture (Figure 1D). In contrast, the opposite side displayed a dense, filamentous surface devoid of any pore structure. A cross-sectional examination revealed a significant presence of interconnected pores among the fibers, and the collagen membrane demonstrated a multilayered porous spatial configuration, potentially due to the increased presence of terminal peptides due to alkaline solution treatment [51].

### 3.3. Porosity Analysis

The average porosity of squid skin decellularised membrane was determined to be about 57.92%. Meanwhile, the electron microscope scans of squid collagen membranes showed that the membranes were porous and regular in appearance and shape, which provided a basis for their use as bioscaffold materials. An earlier study showed [52] that the ideal guided regeneration membrane should have high porosity and large pore size, which in turn expands the range of interaction between the membrane material and the human body fluids or tissues and facilitates the growth of cell adhesion and nutrient entry, thus accelerating the degradation of the material and promoting the formation of newborn bone [53].

### 3.4. Evaluation of Swelling Performance

**Figure 2:**
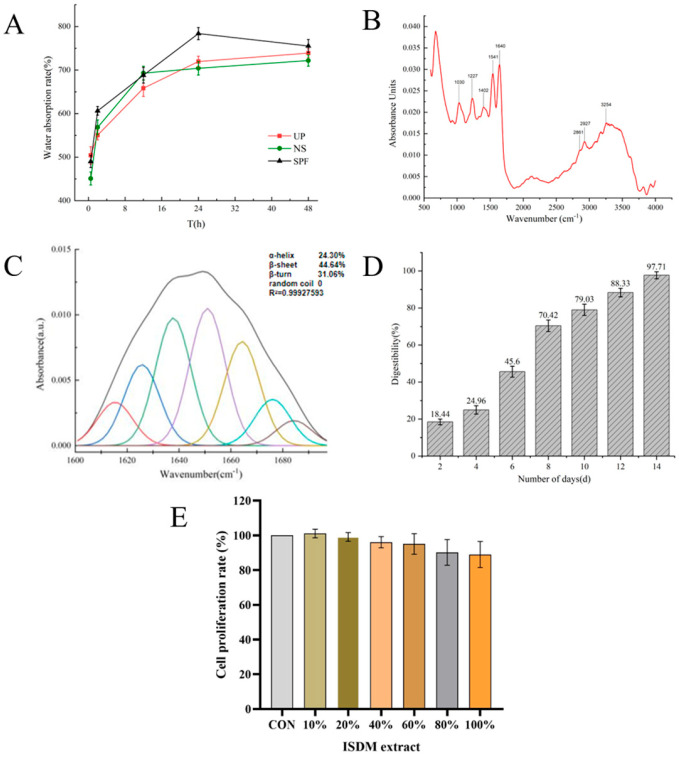
Physical and chemical indicators testing. (**A**) Solubility of SADM in different solutions (ultrapure water, saline, SPF simulated tissue fluid); (**B**) SADM’s Fourier transform infrared spectrum (FTIR); (**C**) The secondary structure of SADM was determined by Gaussian deconvolution analysis of the amide I region; (**D**) In vitro enzyme digestion rate of SADM; (**E**) Cell proliferative effects of ISDM extract in MSCs cells.

The dissolution rate of the decellularized squid skin membrane (Figure 2A) demonstrated a water absorption of 504.1 ± 10.1% at 0.5 h. This absorption rate subsequently decreased to 719.8 ± 14.4% at 24 h and 739.0 ± 14.8% at 48 h. Beyond the 24-h mark, the water absorption rate gradually increased and eventually stabilized, reaching saturation. This behavior is likely attributed to the presence of hydrophilic groups within the collagen membrane, along with its triple helix structure, which exhibits significant water retention capabilities. Effective water absorption is crucial for biomedical materials; inadequate absorption of initial blood seepage during guided bone regeneration using collagen-based membranes can lead to surgical complications [54,55]. The findings indicate that decellularized membranes derived from squid skin possess remarkable hydrophilicity and water retention characteristics [56], effectively supporting the internal environment necessary for tissue or cell proliferation.

### 3.5. Fourier Infrared Spectral Analysis

The infrared spectra of the samples presented in Figure 2B and Table 1 indicate that the peak at 3254 cm^−1^ is associated with O-H and N-H stretching vibrations [57]. The peaks at 2927 cm^−1^ and 2861 cm^−1^ are attributed to CH_2_ stretching vibrations [58]. Additionally, the bands at 1640 cm^−1^, 1541 cm^−1^, and 1227 cm^−1^ correspond to the amide I, II, and III regions of the proteins, respectively. The peak at 1402 cm^−1^ is linked to C-H bending vibrations, while the peak at 1030 cm^−1^ is related to C-O-C stretching vibrations [59]. The secondary structure analysis of the decellularized dermal matrix derived from squid skin, as illustrated in (Figure 2C), revealed that the α-helix content was 24.30%, the β-fold content was 44.64%, and the β-flip content was 31.06%. Additionally, the collagen structure did not exhibit any irregular curls. However, the absence of irregular curl structure was not observed in the collagen, potentially attributed to the higher glycine content inherent in collagen. These findings indicate that the various preparation methods employed for the decellularized squid skin dermal matrix did not influence the secondary structure of collagen [60].

### 3.6. Measurement of Mechanical Properties

The mechanical characteristics of the decellularized membrane derived from squid skin were evaluated through tensile mechanics experiments. The findings indicated a tensile strength of 10.43 ± 0.18 MPa and an elongation at a break of 14.18 ± 0.23%. During the application of tissue engineering materials, they need to be cut to fit the size and shape of the injury area. Therefore, good tensile strength and mechanical properties not only facilitate further processing of collagen scaffold materials but also provide sufficient structural support to resist external pressure from the surrounding tissue [61].

### 3.7. In Vitro Degradation Rate Analysis

The in vitro degradation rate of the decellularized squid skin membrane (Figure 2D) was assessed throughout the degradation process. On day 4, the degradation rate was measured at 24.96 ± 0.92%. By day 8, this rate increased to 70.42 ± 2.1%, accompanied by noticeable shrinkage of the membrane and a slight turbidity in the extracted solution. On day 12, the degradation rate reached about 88.33 ± 1.66%, and the membrane had broken down into flocculent particles, with the extract exhibiting significant turbidity on day 14. For biomaterials primarily composed of collagen, the digestibility of collagenase at a specific concentration in vitro can provide insights into the material’s degradation and absorption capabilities in vivo.

During guided tissue regeneration, the degradation rate of the membrane should align with the tissue growth rate. An excessively rapid degradation can compromise the membrane’s structural integrity, ultimately diminishing the mechanical strength of the bone defect. This rapid breakdown may lead to the swift release of degradation products, such as oligopeptides and amino acids, into the defect area, potentially hindering the regeneration of new bone tissue. Conversely, if the membrane degrades too slowly, prolonged presence in the body may trigger adverse reactions. Therefore, optimal degradation performance is essential to maintain the spatial integrity of the bone defect while preventing the premature entry of degradation products into the organism, thereby facilitating tissue regeneration in the affected area.

### 3.8. Cell Proliferation and Cytotoxicity

**Figure 3:**
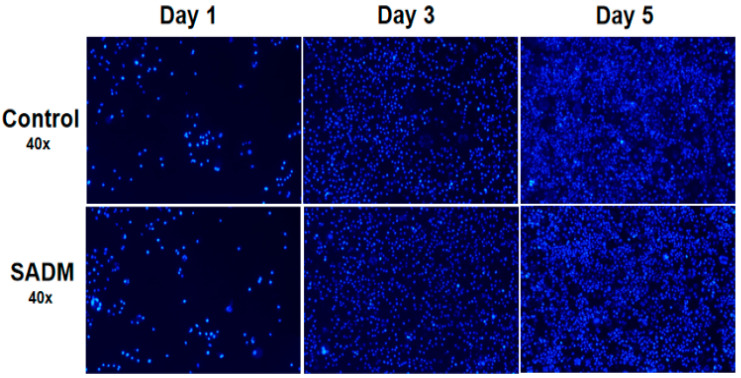
SADM extract to culture MSCs cells on the first, third and fifth day of DAPI staining.

The CCK-8 assay provides a direct assessment of live cell counts, with absorbance readings positively correlating to cell viability. As shown in Figure 2E, the findings indicate that all five experimental groups, which were treated with different concentrations of SADM extracts, achieved a cell survival rate of over 85% in comparison to the control group. Notably, the groups treated with concentrations between 10% and 60% reached cell proliferation rates approaching or surpassing 96%. This low level of cytotoxicity is a significant factor that underscores the potential application of squid skin decellularized membranes. Cytotoxic agents can negatively impact cellular metabolism, as demonstrated by DAPI staining, which highlights blue nuclei within the cells. Figure 3 illustrates a notable trend in proliferation for both the control and SADM groups at 3 and 5 days. Importantly, the SADM group exhibited a more favorable growth trend compared to the control group, with no significant alterations in cell morphology, a minimal presence of dead cells, and no substantial differences in the percentage of dead cells. This indicates that SADM extracts facilitate MSC proliferation while preserving cell integrity [62].

### 3.9. Characteristics of SADM Repair of Bone Cavity Defects in the Rabbit Cranial Parietal Bone

#### 3.9.1. Behavioural Characteristics of Rabbits with Cranial Cave-In Bone Defects

The experimental animals were healthy and survived until the completion of the experiment. Thirty minutes after the operation, the experimental animals were completely alert; however, their food intake and activity levels were diminished. They resumed normal eating habits for three days post-surgery, and there were no signs of redness, swelling, or inadequate healing [63].

#### 3.9.2. Effect of SADM on New Osteogenesis in Rabbit Skulls

**Figure 4:**
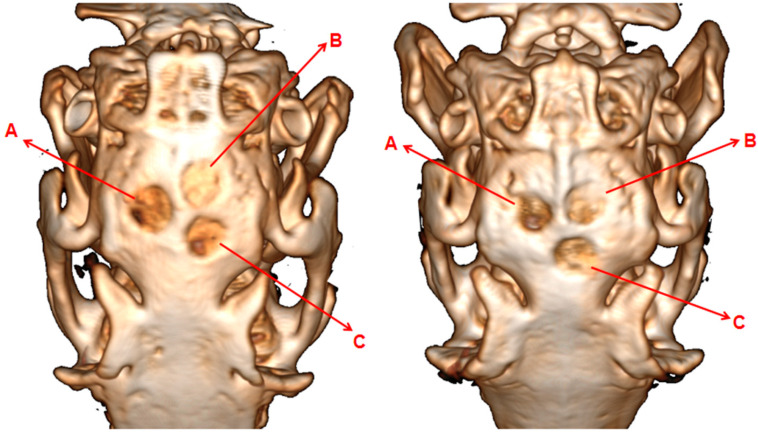
Microscopic CT observations of the animal model at postoperative weeks 4 (**Left**) and 8 (**Right**). The red arrows indicate A: Blank Group, B: Squid Collagen Membrane, C: HEAL-ALL Membrane).

The bone defect was distinctly visible, with a clear demarcation from the adjacent bone tissue in the blank group after four weeks of surgery (Figure 4). In the experimental group, the defect area showed significant new bone formation, resulting in a blurred boundary with the surrounding bone tissue, and the edge of the bone scab appeared hazy. The positive control group exhibited new bone replacing the defect area; however, a defect foramen remained, and the edge of the bone scab was also hazy. At the same time, the blank group still displayed a noticeable defect area, with slow new bone growth in eight weeks of post-surgery (Figure 4). In the experimental group, compared to week four, there was marked resorption of the membrane material and evident regeneration of bone tissue. Most of the defect area was filled with new bone, the boundary with the surrounding tissue was significantly less distinct, and the volume of bone scab exceeded half of the defect area, aligning closely with the surrounding bone. The positive control group showed that the original defect area was no longer porous compared to week four, with the bone scabs largely filling the defect area and connecting to the cortical bone, although the defect area itself remained unfilled. This finding indicates that during the same repair timeframe, collagen membranes play a more crucial role in enhancing bone tissue regeneration and can more efficiently expedite the healing process of bone tissue. Additionally, the experimental findings have confirmed the excellent biocompatibility of collagen membranes, which are fully absorbable by the surrounding tissue.

#### 3.9.3. Effects of SADM on New Osteogenesis in Rabbit Cranium Observed in Saffron Solid Green Sections

**Figure 5:**
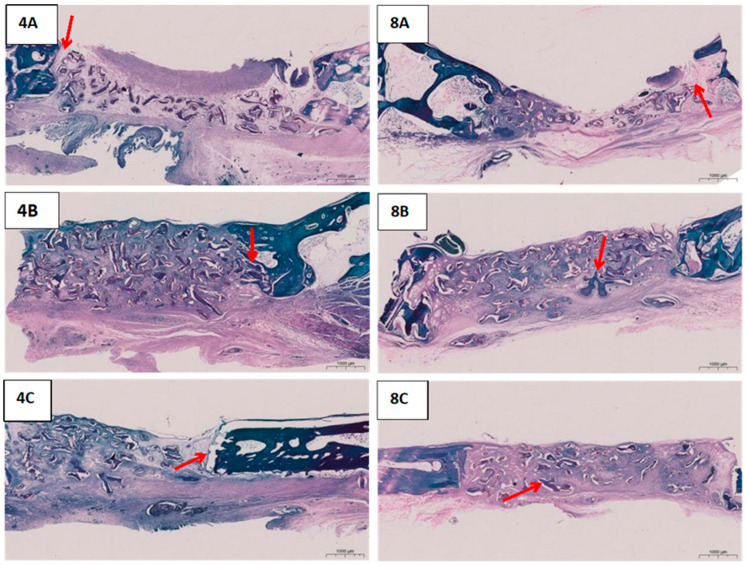
Safranin green staining observation of post-operative animal models. A: Blank Group B: Squid Collagen Membrane C: HEAL-ALL Membrane. The numbers 4 and 8 represent weeks 4 and 8, respectively. Red arrows indicate the presence of new and mature bone trabeculae in the bone defect area.

The blank group had bone tissue that exhibited an abnormal overall structure, with visible new bone trabeculae and a significant presence of fibrous tissue in the defect area in four weeks of post-surgery (Figure 5). The distinction between osteogenic bone and new bone trabeculae was clearly defined (indicated by the red arrow in Figure 5). Experimental group: the bone tissue structure was slightly abnormal, showing new bone trabeculae in the defect area, but the boundary between osteogenic bone and new bone trabeculae was not well-defined (as indicated by the red arrows in Figure 5). Positive control group: the overall structure of the bone tissue was mildly abnormal, with new bone trabeculae present in the defect area, and the boundary between osteogenic and new bone trabeculae was clearly visible.

The overall structure of the bone tissue remained abnormal, with new bone trabeculae and a limited amount of fibrous tissue observed in the defect area, where the borders between mature bone and new bone trabeculae were distinctly clear in the Blank group after eight weeks of surgery (as shown by red arrows in Figure 5).

In the experimental group, the bone tissue structure was mildly abnormal, displaying both new and more mature bone trabeculae in the defect area (as indicated by the red arrows in Figure 5). In the Positive control group, the overall structure of the bone tissue was mildly abnormal, with new bone trabeculae and a small number of mature bone trabeculae evident in the defect area. After surgery, the experimental group exhibited the characteristic of blurred boundaries between osteogenesis and new bone trabeculae, while the blank group and the positive control group had clear boundaries. Combined with the histological features, it was confirmed that the collagen membrane material could accelerate the fusion of new bone and existing bone tissue by promoting the migration of osteoblasts or accelerating the mineralization of bone matrix. This phenomenon is similar to the bone integration process mediated by bone morphogenetic protein (BMP). This result provides key evidence for the development of new bone repair materials.

In the histological analysis, the main filling tissue in the bone defect area, apart from the neoplastic bone tissue, was fibrous tissue: a clear filling of fibrous tissue was observed in the bone defect area in the blank group (not covered with any material) at both 4 and 8 weeks postoperatively (Figure 5). These fibrous tissues were clearly demarcated from the new bone trabeculae, indicating that in the absence of effective guidance, the defect area was mainly occupied by fibrous connective tissue, which hindered the bone regeneration process. In addition, a neovascular network was formed to support the mineralization and maturation of bone tissue. The collagen matrix and active components (e.g., amino acids, cytokines, etc.) released during degradation of the material in the experimental group (SADM) and positive control group (HEAL-ALL membrane) provided microenvironmental support for cell migration and bone regeneration. The porous structure of the material (SADM porosity of 58%) promotes cellular infiltration and nutrient exchange, indirectly regulating local inflammatory responses and osteogenic differentiation. There was no significant difference in the osteogenic capacity between the experimental group (SADM) and the positive control group (HEAL-ALL membrane) as barrier membranes, but the experimental group (SADM) had a higher bone maturation.

#### 3.9.4. Histometric Findings

**Figure 6:**
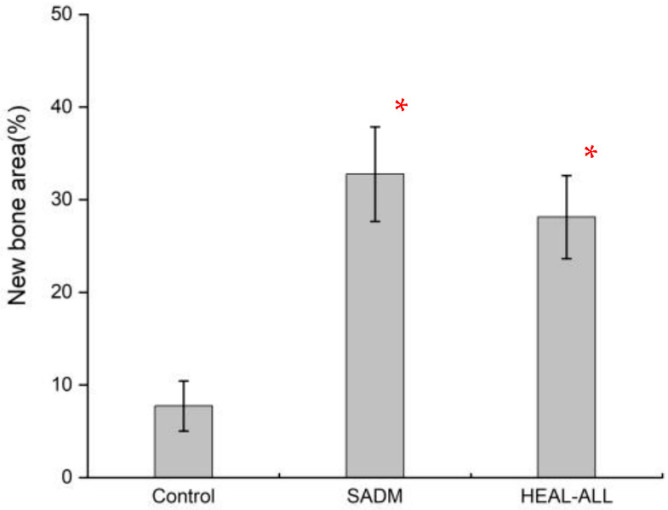
Histometric measurement of newly formed bones (* *p* < 0.05).

The mean and standard deviations of the new bone area are shown in Table 2 and Figure 6. At 8 weeks, the new bone area (%) of the Control, SADM, and HEAL-ALL groups were 7.73 (±2.71), 32.76 (±5.11), and 28.12 (±4.48), respectively. The HEAL-ALL groups and SADM groups showed significant differences from the Control (*p* > 0.05).

## 4. Conclusions

In this study, we successfully developed and characterized tissue regeneration membranes, specifically focusing on the impact of squid skin-derived collagen membranes (SADM) in guided bone regeneration techniques for repairing cranial cavity-type bone defects in rabbits. The SADM was created through a meticulous process involving physical and thermal cross-linking following decolorization, degreasing, and decellularization. Our findings indicate that SADM exhibits excellent porosity and mechanical strength while remaining non-toxic to cells, fulfilling the essential criteria for biomedical materials. The results from our animal experiments demonstrate that SADM effectively facilitates the migration of osteoblasts into the bone defect area, thereby promoting new bone formation. Furthermore, the squid skin tissue regeneration membrane not only meets the fundamental requirements for biomedical applications but also shows significant potential in enhancing skin wound healing. Given these promising outcomes, SADM presents a viable option for further development as a scaffold material for medical bone regeneration, indicating strong prospects for future applications in regenerative medicine.

## Figures and Tables

**Table 1:** FTIR spectral peak positions and attribution of squid decellularised membranes (SADM).

Peaks	Absorption [cm^−1^]	Observations
Amide A	3254	O-H N-H Tension
Amide B	2927–2861	CH_2_ asymmetric stretching
Amide I	1640	C=O stretch Hydrogen bonding and COO-coupling
Amide II	1541	N-H bending coupled to CN stretching
Amide III		

**Table 2:** Mean and standard deviation (SD) of new bone area (n = 3).

	Groups	Mean	SD	*p*-Value
New bone area (%)	Control	7.73	2.71	0.017 *
	SADM	32.76	5.11	
	HEAL-ALL	28.12	4.48	

* *p* < 0.05.

## Data Availability

The raw data supporting the conclusions of this article will be made available by the corresponding authors on request.

## References

[1] Xu S.T., Ge B.F., Xu Y.K. (2005). Practical Orthopaedics.

[2] Park S.A., Shin J.W., Yang Y.I., Kim Y.K., Park K.D., Lee J.W., Jo I.H., Kim Y.J. (2004). In vitro study of osteogenic differentiation of bone marrow stromal cells on heat-treated porcine trabecular bone blocks. Biomaterials.

[3] Kasaj A., Reichert C., Götz H., Röhrig B., Smeets R., Willershausen B. (2008). In vitro evaluation of various bioabsorbable and nonresorbable barrier membranes for guided tissue regeneration. Head Face Med..

[4] Brotto M., Johnson M.L. (2014). Endocrine Crosstalk Between Muscle and Bone. Curr. Osteoporos. Rep..

[5] Frost H.M. (2010). From Wolff’s law to the Utah paradigm: Insights about bone physiology and its clinical applications. Anat. Rec..

[6] Manolagas S.C. (2000). Birth and death of bone cells: Basic regulatory mechanisms and implications for the pathogenesis and treatment of osteoporosis. Endocr. Rev..

[7] Dahlin C., Sennerby L., Lekholm U., Linde A., Nyman S. (1989). Generation of new bone around titanium implants using a membrane technique: An experimental study in rabbits. Int. J. Oral Maxillofac. Implant..

[8] Rakers S., Gebert M., Uppalapati S., Meyer W., Maderson P., Sell A.F., Kruse C., Paus R. (2010). ‘Fish matters’: The relevance of fish skin biology to investigative dermatology. Exp. Dermatol..

[9] Elango J., Robinson J., Zhang J., Bao B., Ma N., De Val J.E.M.S., Wu W. (2019). Collagen Peptide Upregulates Osteoblastogenesis from Bone Marrow Mesenchymal Stem Cells through MAPK-Runx2. Cells.

[10] Banerjee P., Mehta A., Shanthi C. (2014). Investigation into the cyto-protective and wound healing properties of cryptic peptides from bovine Achilles tendon collagen. Chem. Interact..

[11] Wong R., Rabie A. (2010). Effect of Bio-Oss Collagen and Collagen matrix on bone formation. Open Biomed. Eng. J..

[12] Alam K., Jeffery S.L.A. (2019). Acellular Fish Skin Grafts for Management of Split Thickness Donor Sites and Partial Thickness Burns: A Case Series. Mil. Med..

[13] Lim Y.S., Ok Y.J., Hwang S.Y., Kwak J.Y., Yoon S. (2019). Marine Collagen as A Promising Biomaterial for Biomedical Applications. Mar. Drugs.

[14] Sapru S., Das S., Mandal M., Ghosh A.K., Kundu S.C. (2021). Sericin-chitosan-glycosaminoglycans Hydrogels Incorporated with Growth Factors for In Vitro and In Vivo Skin Repair. Carbohydr. Polym..

[15] Zhou H., Shang L., Li X., Zhang X., Gao G., Guo C., Chen B., Liu Q., Gong Y., Shao C. (2009). Resveratrol augments the canonical Wnt signaling pathway in promoting osteoblastic differentiation of multipotent mesenchymal cells. Exp. Cell Res..

[16] Dai Z., Li Y., Quarles L.D., Song T., Pan W., Zhou H., Xiao Z. (2007). Resveratrol enhances proliferation and osteoblastic differentiation in human mesenchymal stem cells via ER-dependent ERK1/2 activation. Phytomedicine.

[17] Mao A.S., Mooney D.J. (2015). Regenerative medicine: Current therapies and future directions. Proc. Natl. Acad. Sci. USA.

[18] Kjartansson H., Olafsson I.H., Karason S., Thorisson H., Baldursson B.T., Gunnarsson E., Jorundsson E., Sigurjonsson G.F. (2015). Use of Acellular Fish Skin for Dura Repair in an Ovine Model: A Pilot Study. Open J. Mod. Neurosurg..

[19] Dorweiler B., Trinh T.T., Dünschede F., Vahl C.F., Debus E.S., Storck M., Diener H. (2017). Die marine Omega-3-Wundmatrix zur Behandlung komplizierter Wunden. Gefasschirurgie.

[20] Zhang Q., Wang Q., Lv S., Lu J., Jiang S., Regenstein J.M., Lin L. (2016). Comparison of collagen and gelatin extracted from the skins of Nile tilapia (*Oreochromis niloticus*) and channel catfish (*Ictalurus punctatus*). Food Biosci..

[21] Cho J.-K., Jin Y.-G., Rha S.-J., Kim S.-J., Hwang J.-H. (2014). Biochemical characteristics of four marine fish skins in Korea. Food Chem..

[22] Jafari H., Lista A., Siekapen M.M., Ghaffari-Bohlouli P., Nie L., Alimoradi H., Shavandi A. (2020). Fish Collagen: Extraction, Characterization, and Applications for Biomaterials Engineering. Polymers.

[23] Cen L., Liu W., Cui L., Zhang W., Cao Y. (2008). Collagen Tissue Engineering: Development of Novel Biomaterials and Applications. Pediatr. Res..

[24] Loss M., Wedler V., Künzi W., Meuli-Simmen C., Meyer V.E. (2000). Artificial Skin, Split-Thickness Autograft and Cultured Autologous Keratinocytes Combined to Treat a Severe Burn Injury of 93% of TBSA. Burns.

[25] Fitton A.R., Drew P., Dickson W.A. (2001). The Use of a Bilaminate Artificial Skin Substitute (IntegraTM) in Acute Resurfacing of burns: An Early Experience. Br. J. Plast. Surg..

[26] Crapo P.M., Gilbert T.W., Badylak S.F. (2011). An Overview of Tissue and Whole Organ Decellularisation Processes. Biomaterials.

[27] Nyström A., Bruckner-Tuderman L. (2019). Matrix Molecules and Skin Biology. Semin. Cell Dev. Biol..

[28] Li D., Sun W.Q., Wang T., Gao Y., Wu J., Xie Z., Zhao J., He C., Zhu M., Zhang S. (2021). Evaluation of a novel tilapia-skin acellular dermis matrix rationally processed for enhanced wound healing. Mater. Sci. Eng. C-Mater. Biol. Appl..

[29] Fosnot J., Kovach S.J., Serletti J.M. (2011). Acellular Dermal Matrix:General Principles for the Plastic Surgeon. Aesthetic Surg. J..

[30] Wang L.-P., Wang H.-J., Hou X.-S., Raza A., Koyama Y., Ito T., Wang J.-Y. (2021). Preparation of stretchable composite film and its application in skin burn repair. J. Mech. Behav. Biomed. Mater..

[31] Stone R., Saathoff E.C., Larson D.A., Wall J.T., Wienandt N.A., Magnusson S., Kjartansson H., Natesan S., Christy R.J. (2021). Accelerated Wound Closure of Deep Partial Thickness Burns with Acellular Fish Skin Graft. Int. J. Mol. Sci..

[32] Chen J., Gao K., Liu S., Wang S., Elango J., Bao B., Dong J., Liu N., Wu W. (2019). Fish Collagen Surgical Compress Repairing Characteristics on Wound Healing Process In Vivo. Mar. Drugs.

[33] Hu Z., Yang P., Zhou C., Li S., Hong P. (2017). Marine Collaen Peptides from the Skin of Nile Tilapia (*Oreochromis niloticus*). Characterisation and Wound Healing Evaluation. Mar. Drugs.

[34] Stähli A., Miron R.J., Bosshardt D.D., Sculean A., Gruber R. (2016). Collagen membranes adsorb the transforming growth factor-β receptor I kinase-dependent activity of enamel matrix derivative. J. Periodontol..

[35] Sarban S., Senkoylu A., Isikan U.E., Korkusuz P., Korkusuz F. (2009). Can rhBMP-2 containing collagen sponges enhance bone repair in ovariectomised rats?: A preliminary study. Clin. Orthop. Relat. Res..

[36] Yan W., Chen S. (2013). Analysis of squid production and import/export trade in China. China Fish. Econ..

[37] Nam K.A., You S.G., Kim S.M. (2008). Molecular and physical characteristics of squid (Todarodes pacificus) skin collagens and biological properties of their enzymatic hydrolysates. J. Food Sci..

[38] Nakchum L., Kim S.M. (2016). Preparation of squid skin collagen hydrolysate as an antihyaluronidase, antityrosinase, and antioxidant agent. Prep. Biochem. Biotechnol..

[39] Mutalipassi M., Esposito R., Ruocco N., Viel T., Costantini M., Zupo V. (2021). Bioactive Compounds of Nutraceutical Value from Fishery and Aquaculture Discards. Foods.

[40] Dorati R., Colonna C., Genta I., Modena T., Conti B. (2010). Effect of porogen on the physico-chemical properties and degradation performance of PLGA scaffolds. Polym. Degrad. Stab..

[41] Amirian J., Zeng Y., Shekh M.I., Sharma G., Stadler F.J., Song J., Du B., Zhu Y. (2021). In-situ crosslinked hydrogel based on amidated pectin/oxidized chitosan as potential wound dressing for skin repairing. Carbohydr. Polym..

[42] Zhang D., Hou J., Gu Y., Shao J., Zhou S., Zhuang J., Song L., Wang X. (2021). Cryopreserved skin epithelial cell sheet combined with acellular amniotic membrane as an off-the-shelf scaffold for urethral regeneration. Mater. Sci. Eng. C.

[43] Tan J., Li L., Wang H., Wei L., Gao X., Zeng Z., Liu S., Fan Y., Liu T., Chen J. (2021). Biofunctionalized fibrin gel co-embedded with BMSCs and VEGF for accelerating skin injury repair. Mater. Sci. Eng. C.

[44] Wang S., He S., Zhang X., Sun J., Huang Q., Liu J., Han C., Yin Z., Ding B., Yin J. (2021). Acellular bovine pericardium matrix in immediate breast reconstruction compared with conventional implant-based breast reconstruction. JPRAS Open.

[45] Sorushanova A., Delgado L.M., Wu Z., Shologu N., Kshirsagar A., Raghunath R., Mullen A.M., Bayon Y., Pandit A., Raghunath M. (2019). The Collagen Suprafamily: From Biosynthesis to Advanced Biomaterial Development. Adv. Mater..

[46] Ferreira A.M., Gentile P., Chiono V., Ciardelli G. (2012). Collagen for bone tissue regeneration. Acta Biomater..

[47] Zhao C., Xiao Y., Ling S., Pei Y., Ren J. (2021). Structure of Collagen. Methods Mol. Biol..

[48] Sivaraman K., Muthukumar K., Shanthi C. (2022). Adhesion and proliferation properties of type I collagen-derived peptide for possible use in skin tissue engineering application. Cell Biol. Int..

[49] Schmitz J.P., Hollinger J.O. (1986). The critical size defect as an experimental model for craniomandibulofacial nonunions. Clin. Orthop. Relat. Res..

[50] Norton M.R., Odell E.W., Thompson I.D., Cook R.J. (2003). Efficacy of bovine bone mineral for alveolar augmentation: A human histologic study. Clin. Oral Implant. Res..

[51] Jaziri A.A., Shapawi R., Mokhtar R.A.M., Noordin W.N.M., Huda N. (2022). Biochemical and Microstructural Properties of Lizardfish (*Saurida tumbil*) Scale Collagen Extracted with Various Organic Acids. Gels.

[52] Beard H.K., Ueda M., Faulk W.P., Glynn L.E. (1978). Cell-mediated and humoral immunity to chick type II collagen and its cyanogen bromide peptides in guinea-pigs. Immunology.

[53] Duan Z. (2008). Research on the Properties of Humanoid Collagen Haemostatic Sponge. Ph.D. Thesis.

[54] Hutmacher D., Hürzeler M.B., Schliephake H. (1996). A review of material properties of biodegradable and bioresorbable polymers and devices for GTR and GBR applications. Int. J. Oral Maxillofac. Implant..

[55] Pati F., Datta P., Adhikari B., Dhara S., Ghosh K., Das Mohapatra P.K. (2012). Collagen scaffolds derived from fresh water fish origin and their biocompatibility. J. Biomed. Mater. Res. Part A.

[56] Doyle B.B., Bendit E.G., Blout E.R. (1975). Infrared Spectroscopy of Collagen and Collagen-like Polypeptides. Biopolymers.

[57] Abe Y., Krimm S. (1972). Normal Vibrations of Crystalline Polyglycine I. Biopolymers.

[58] Payne K.J., Veis A. (1988). Fourier Transform Ir Spectroscopy of Collagen and Gelatin Solutions: Deconvolution of the Amide I Band for Conformational Studies. Biopolymers.

[59] Wabnitz G.H., Goursot C., Jahraus B., Kirchgessner H., Hellwig A., Klemke M., Konstandin M.H., Samstag Y. (2010). Mitochondrial Translocation of Oxidized Cofilin Induces Caspase-Independent Necrotic-like Programmed Cell Death of T Cells. Cell Death Dis..

[60] Ma Y., Chu S., Sun Y., Ma S., Li X., Zhang T., Zhou Y. (2015). Preparation of poly(butylene succinate)/poly(propylene carbonate) biofilm and evaluation of related properties. China Tissue Eng. Res..

[61] Park S., Choi S., Shimpi A.A., Estroff L.A., Fischbach C., Paszek M.J. (2024). Collagen Mineralization Decreases NK Cell-Mediated Cytotoxicity of Breast Cancer Cells via Increased Glycocalyx Thickness. Adv. Mater..

[62] Ellis M.W., Riaz M., Huang Y., Qyang Y.B. (2022). Epigallocatechin Gallate Facilitates Extracellular Elastin Fiber Formation in Induced Pluripotent Stem Cell Derived Vascular Smooth Muscle Cells for Tissue Engineering. J. Mol. Cell. Cardiol..

[63] Eyrebrook A.L. (1984). The periosteum: Its function reassessed. Clin. Orthop. Relat. Res..

